# ATP synthesis is active on the cell surface of the shrimp *Litopenaeus vannamei* and is suppressed by WSSV infection

**DOI:** 10.1186/s12985-015-0275-7

**Published:** 2015-03-29

**Authors:** Yan Liang, Meng-Lin Xu, Xiao-Wen Wang, Xiao-Xiao Gao, Jun-Jun Cheng, Chen Li, Jie Huang

**Affiliations:** Key Laboratory of Sustainable Development of Marine Fisheries, Ministry of Agriculture, Yellow Sea Fisheries Research Institute, Chinese Academy of Fishery Sciences, No.106 Nanjing Road, Qingdao, 266071 China

**Keywords:** White spot syndrome virus, Shrimp, F_1_-ATP synthase beta subunit, Cell surface ATP synthesis, Virus binding, RNAi, Receptor

## Abstract

**Background:**

Over the past a few years, evidences indicate that adenosine triphosphate (ATP) is an energy source for the binding, maturation, assembly, and budding process of many enveloped viruses. Our previous studies suggest that the F_1_-ATP synthase beta subunit (ATPsyn β, BP53) of the shrimp *Litopenaeus vannamei* (*L. vannamei*) might serve as a potential receptor for white spot syndrome virus (WSSV)’s infection.

**Methods:**

BP53 was localized on the surface of shrimp hemocytes and gill epithelial cells by immunofluorescence assay and immunogold labeling technique. Cell surface ATP synthesis was demonstrated by an *in vitro* bioluminescent luciferase assay. Furthermore, the expression of *bp53* after WSSV infection was investigated by RT-PCR test. In addition, RNAi was developed to knock down endogenous *bp53*.

**Results:**

BP53 is present on shrimp cell surface of hemocytes and gill epithelia. The synthesized ATP was detectable in the extracellular supernatant by using a bioluminescence assay, and the production declined post WSSV binding and infection. Knocking down endogenous *bp53* resulted in a 50% mortality of *L. vannamei*.

**Conclusion:**

These results suggested that BP53, presenting on cell surface, likely served as one of the receptors for WSSV infection in shrimp. Correspondingly, WSSV appears to disturb the host energy metabolism through interacting with host ATPsyn β during infection. This work firstly showed that host ATP production is required and consumed by the WSSV for binding and proceeds with infection process.

## Introduction

White spot syndrome virus (WSSV), the only member of the genus *Whispovirus* of the family of *Nimaviridae*, has emerged globally as one of the most prevalent and lethal pathogen for Penaeid shrimp species since its first outbreak in 1992 [1]. The better understanding of its pathogenesis, especially the nature of virus–host interactions, will eventually lead to the development of new strategies to control white spot viral disease. It is well known that attachment/binding to the host cell surface is essential for initiation of a viral infection [2]. This virus–host interactions may also trigger a serial host immune responses against the invader as well as some modifications of host gene expression to facilitate virus replication [3]. Recently several WSSV envelope proteins [VP37 (VP281), VP28, VP187, and VP53A], and some cellular proteins of shrimp [PmRab7, β-integrin, PmCBP, and F_1_-ATP synthase β subunit (ATPsyn β, also named BP53)] have been reported in relation to the process of virus particles attachment and entry into host cells [4-9]. However, it is still unclear about how viral infections cause profound alterations in host cells [7].

ATPsyn β, as part of F_1_Fo ATP synthase complexes, was originally described in the inner membrane of mitochondria. However, ATPsyn β have been found on the surface of tumor cells, and serves as a receptor of angiostatin [10]. In 2009, ATPsyn β was also reported on the surface of Hpt cells from crayfish *Pacifastacus leniusculus* (*P. leniusculus*). It was identified as a receptor for the invertebrate cytokine astakine and is involved in hematopoiesis [10]. In our previous study, we identified an ATPsyn β (named as BP53, GenBank accession number EU401720) in shrimp, which served as a receptor for WSSV binding [8]. Further study indicated that the viral virulence can be attenuated after mixing the WSSV with recombinant (r) BP53 [8]. This is the the first work aimed to elucidate the role of shrimp ATPsyn β with WSSV infection. In 2012, VP37, an important structural protein of WSSV was also demonstrated to be able to binding with ATPsyn β in another lab. Their results also revealed that three anti-ATPsyn β monoclonal antibodies could partially block the binding of WSSV to shrimp both *in vitro* and *in vivo* [11]. Based on these studies, we speculate that BP53 might act as a candidate receptor of WSSV.

The objectives of the present study were 1) to probe whether BP53 is present on the hemocyte and gill cell surface of shrimp as candidate receptor for WSSV infection, and 2) to investigate its biological function during WSSV infection.

## Results

### BP53 polyclonal antibody preparation and specificity characterization

**Figure 1:**
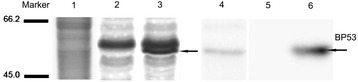
**Specificity Characterization of the polyclonal antibody against BP53 by western blot.** Line marker, pre-stained protein molecular mass markers (MBI, USA); Line 1 to Line 3, SDS-PAGE of gill membrane proteins extracted from WSSV-free *Litopenaeus vannamei* (Line 1), the lysates from *E. coli* Top10 cells with blank plasmid pBAD-gIIIB (Line 2, negative control), lysates of E. coli Top10 cells with recombinant plasmid pBAD-gIIIB-BP53 (Line 3). Line 4 to Line 6, identification of BP53 using anti-rBP53 antibody by western blot. Samples loaded were as same as Line 1 to Line 3 in sequence.

After immunization of New Zealand rabbit with rBP53 protein, the antiserum was obtained. The western blot results showed that the polyclonal antibodies could specifically identify BP53 in both recombinant cell lysates and extracted proteins of gill membrane (Figure 1).

### BP53 immunolocalization on shrimp hemocytes cell surface

**Figure 2:**
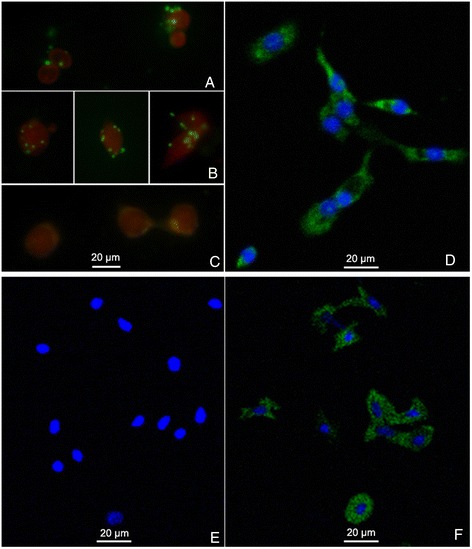
**Localization of BP53 in hemocytes by immunofluorescence assay. (A)** and **(B)**, Normal shrimp hemocytes incubated with anti-BP53 polyclonal antibody, which showed punctate structures distributed over the entire cell surface. **(C)**, Negative control. Normal shrimp hemocytes incubated with pre-immune rabbit serum instead of anti-BP53 polyclonal antibody, which had no detectable fluorescence signal. **(D)**, Permeabilized shrimp hemocytes incubated with anti-BP53 polyclonal antibody, which showed the intracellular expression of BP53 in a characteristic reticular pattern. **(E)**, Normal hemocytes incubated with anti-actin antibody as control group, which didn’t show any positive signals. **(F)**, Permeabilized cells incubated with anti-actin antibody, while showed positive actin signals. Evans blue was used to visualize intact cells, and DAPI was used to visualize nuclei.

The presence of the BP53 on the surface of hemocytes was detected by immunofluorescence with the polyclonal antibodies specific for the β subunit of ATP synthase generated against the recombinant BP53. The immunofluorescence was observed in each cell as one or more irregular clusters of punctate structures (Figure 2A, B), suggesting an organized distribution on the cell surface.

Fluorescence signal on cell surface of Figure 2A, B was determined on the basis of two criteria. (i) Permeabilized cells produced a dramatically different reticular pattern, with a characteristic perinuclear distribution (Figure 2D). (ii) Staining with a known cytoskeleton protein marker actin, produced no detectable signals on intact cells (Figure 2E), while actin staining appeared in cells with enhanced permeability (Figure 2F). This result indicates that anti-BP53 antibodies were binding to the extracellular component of BP53 on the cell surface.

### BP53 immunolocalization on shrimp gill cuticular membrane

**Figure 3:**
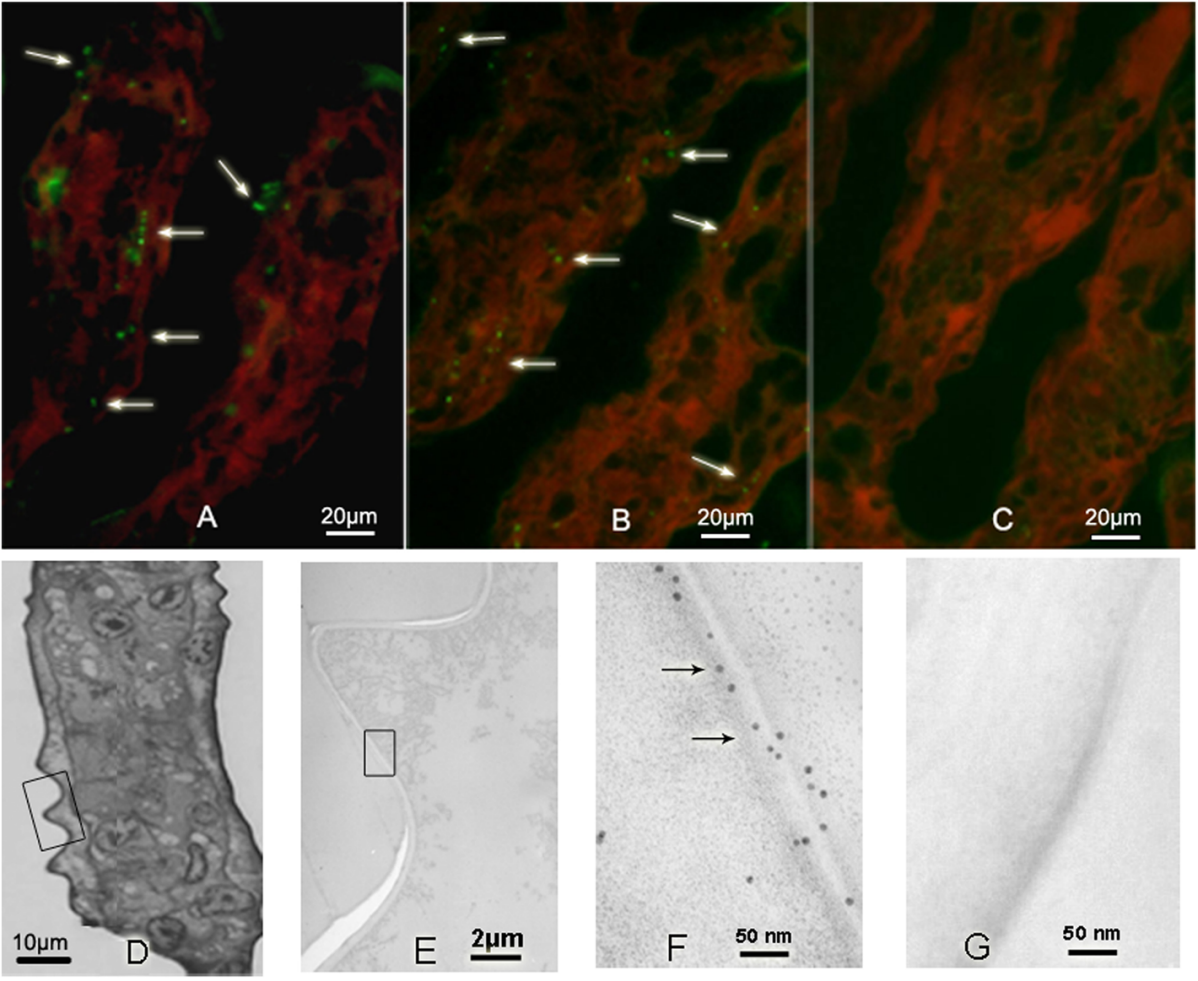
**The localization of BP53 in gill secondary filaments.** The expression of BP53 was analyzed by immunofluorescence microscopy and immunogold electron microscopy, respectively. **(A)** and **(B)**, Gill tissues incubated with anti-BP53 polyclonal antibody, the fluorescence signals were developed by FITC-conjugated HRP, which showed numerous green spots distributed along the cuticular epithelium of gills. Pre-immune serum staining was performed as negative control **(C)**, of which no green fluorescence spot was observed. Evansblue was used to visualize gill tissue, which showed red color under 550 nm laser light. **(D)**, Histological slide of gill tissue observed under microscopy (1000 x), T-E stained. **(E)**, The framed part of picture D was observed under electron microscopy. **(F)**, Enlarged picture of part of the membrane picture E, which showed golden particles appeared along the cell membrane of the epithelium under the cuticle of gills. **(G)**, Negative control, pre-immune serum was used as primary antibody instead of anti-BP53 Ab. Arrows point positive staining.

For subcellular localization of BP53 in the gill tissue, both immunofluorescent assay and immunogold assay were performed. Punctate fluorescent signals were visible in the cuticular epithelium along the gill filament (Figure 3A, B), a tissue that is most susceptible to WSSV. Conversely, no signal was detected in the negative control group (samples incubated with pre-immune serum only) (Figure 3C).

Immunogold assay with Au colloidal nanoparticles conjugated antibodies on ultrathin section of *L. vannamei* gill tissue also showed even distribution of numerous gold nanoparticles along the cellular membrane of gill tissue (Figure 3F), while no gold particles were observed in the negative control group (Figure 3G).

### Cell surface ATP synthesis is active and being inhibited by WSSV infection

**Figure 4:**
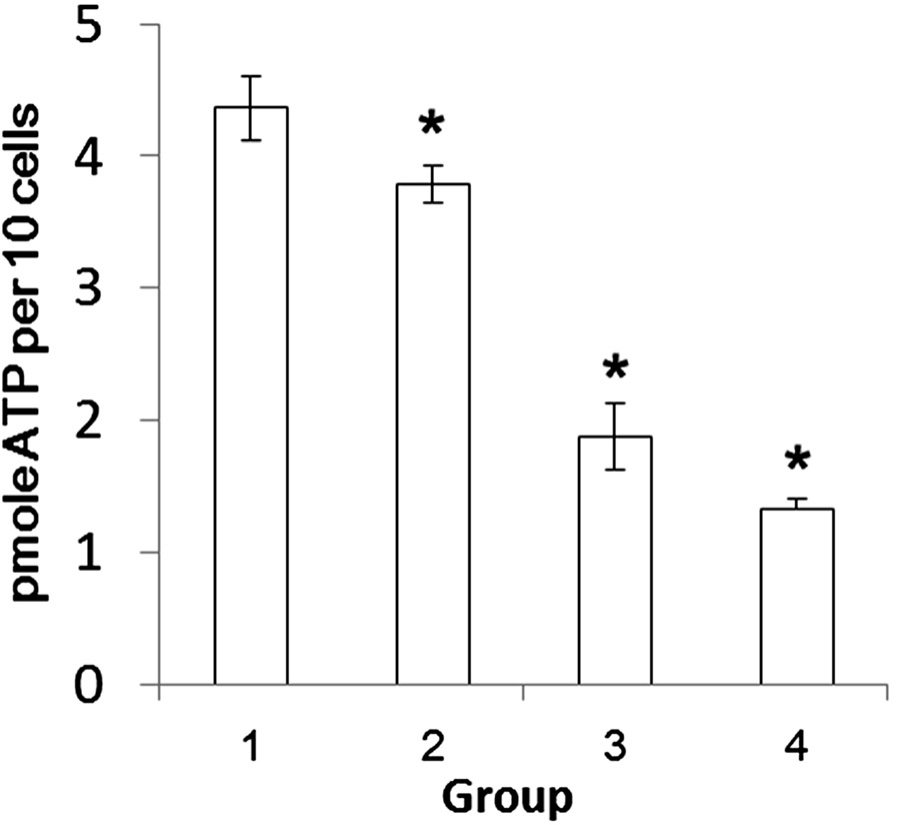
**ATP generation on shrimp hemocytes surface measured by bioluminescent luciferase assay. (1)** ATP production from WSSV free cells, **(2)** ATP production from cells with WSSV bound on the surface, **(3)** ATP production from WSSV naturally infected shrimp cells, **(4)** ATP production from WSSV infected cells that incubated with anti- rBP53-antibody. Asterisk (*) indicates a significant statistical difference between groups (*p* < 0.05).

The F_1_F_O_ ATP synthase holoenzyme efficiently catalyzes both the forward ATP synthase reaction and the reverse ATP hydrolysis reaction. In order to detect ATP synthase activity on the cell surface, the ATP production in the extracellular supernatant was measured using a bioluminescence assay. ATP generation was detected in the presence of ADP and Pi substrates supplemented in the external medium. ATP produced by WSSV-free cells is 4.36 ± 0.24 pmole/10 cells. In contrast, the ATP production was reduced to 42.9% in cells from WSSV infected shrimp (1.87 ± 0.25 pmole/10 cells) (Figure 4). Further, ATP concentration dropped to 86.7% (3.78 ± 0.14 pmole/10 cells) when the cells were incubated with WSSV at 4°C for only 1 h. When rBP53 antibody was incubated with WSSV infected cells, the ATP production was further decreased from 42.9% (1.87 ± 0.25 pmole/10 cells) to 30.5% (1.33 ± 0.07 pmole/10 cells) (*p* < 0.05). No inhibiting effect was observed when rBP53 antibody was incubated with WSSV free cells (Data not shown).

### *bp53* expression responses to WSSV infection

**Figure 5:**
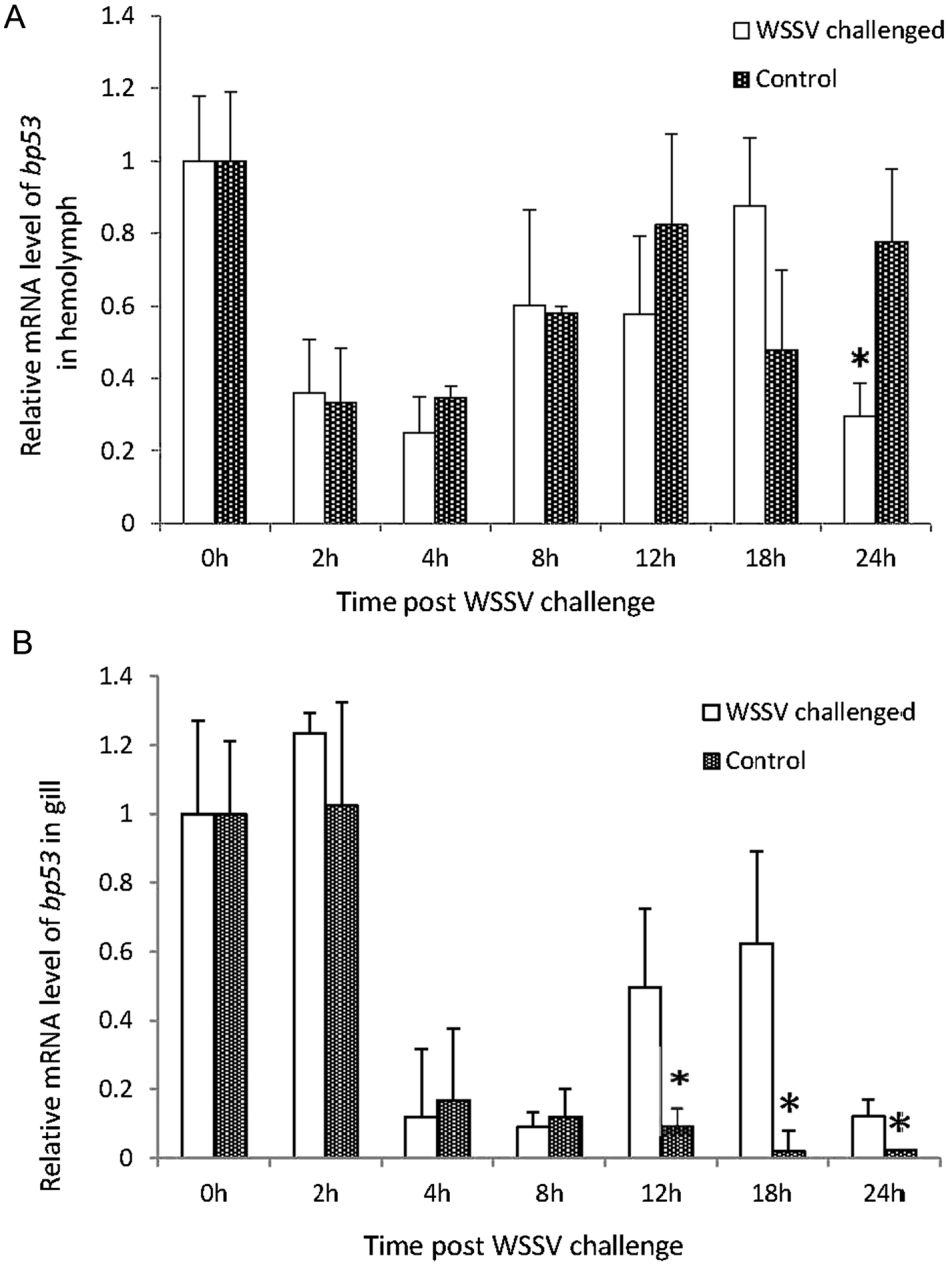
**Time-course of** ***bp53*** **expression in gill and hemolymph after WSSV challenge.** A relative quantitative real-time PCR assay was applied to study *bp53* differential expression profiles in *L. vannamei* hemolymph **(A)** and gill **(B)** in response to WSSV infection within 24 h. Gene expression quantification was determined using the 2^−ΔΔCt^ method. Actin was used as an internal control. Error bars indicate standard deviations (n = 3). Significant differences between the expression level in each time point post injection and the original level were indicated with an asterisk (*p* < 0.05).

Time-course analysis of *bp53* expression was performed after WSSV challenge in 24 hours. Expression profile of *bp53* in hemolymph was shown in Figure 5A. The level of *bp53* expression dramatically decreased in first 4 h post injection, slightly increased at 8 h till 18 h, both in the WSSV challenged and the control group. At 24 h post injection, the expression of *bp53* in the WSSV challenged group decreased significantly (*p* <0.05) in comparison to the gene expression in control group. The transcription levels of *bp53* in gill showed in Figure 5B. The level of *bp53* expression decreased at 4 h post injection and maintained a low level at 8 h in both groups. After 12 h post injection, *bp53* expression increased till the end of the experiment in WSSV challenged group, with significant difference (*p* <0.05) to the gene expression in control group.

### *bp53* is essential for shrimp survival

**Figure 6:**
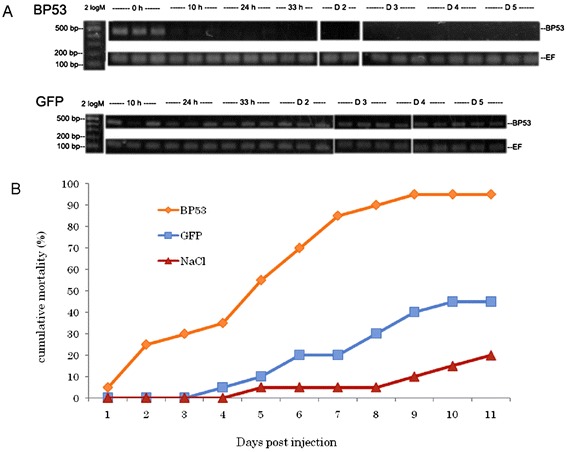
**Effect on shrimp survival after silencing of the** ***bp53*** **gene. (A)**, Endogenous *bp53* mRNA expression after dsRNA injection. BP53 group: *bp53*-specific dsRNA injection; GFP group: *gfp*-specific dsRNA injection. Representative gels of RT-PCR products of *bp53* (482 bp) and *ef* (187 bp, internal control) mRNAs from hemolymph collected at different time points. Label 2 log M indicates the 0.1-10.0 kb DNA ladder marker, while h = hours and D = days post-injection. **(B)**, The cumulative mortality after dsRNA injection without WSSV challenge. BP53 group: *bp53*-specific dsRNA injection; GFP group: *gfp*-specific dsRNA injection; NaCl group: NaCl injection instead of any dsRNA.

The endogenous genes can be knocked down successfully after 10 h post injection of *bp53*-specific dsRNA, and such silencing effect could remain from 33 h up to 5 days post-injection (Figure 6A). Even without WSSV challenge, over 50% of treated shrimp were died by day 5 post-dsRNA injection (Figure 6B), suggesting that BP53 expression is essential for shrimp survival.

## Discussion

Since the first identification of ATP synthase on the surface of cancer cells in 1994 [12] fewer have attempted to characterize its function. We have initially identified an ATPsyn β subunit (named as BP53) as WSSV binding protein in shrimp *Litopenaeus vannamei* [8]. However, the biological functions of ATPsyn β involved in WSSV infection remain unclear. In this study, we extended our initial study and elucidated that BP53 was not only localized inside the cells but was also on the entire cell surface of some hematocytes and the cuticular epithelium membrane of gills, which satisfied the basic requirement to be a candidate receptor for WSSV. Interestingly, involvement of ATPsyn β in the entry of chikungunya virus (CHIKV) into insect cells has been recently demonstrated, and showed a significant reduction in viral entry and virus production by both antibody inhibition and siRNA-mediated down regulation experiments targeted to ATPsyn β [13]. Our current results revealed the evidence that the same protein, ATPsyn β, involved in the infection of WSSV to shrimp, and possibly CHIKV and dengue virus to insect cells as well [13,14]. These informations will lead us a better illustration of the entry process of WSSV into the host cells, as well as the dengue virus into insect cells.

Lin et al. [10] used several different methods to clearly demonstrate that ATP synthase present on the surface of Hpt cells and not on mature blood cells (hemocytes) of crayfish (*P. leniusculus*). In present study, we demonstrated the presence of ATPsyn β on cytoplasm membrane of gill epithelia and some circulating hemocytes in *L. vannamei*. It’s generally agreed that haematopoietic tissue (HPT) is responsible for production and supply of the haemocytes. Hemocytes are synthesised and partly differentiated in the hematopoietic tissue, very little cell proliferation could occurs in the circulation [15]. Besides these, there were three types of mature hemocytes and five immature hemocytes [15-17], so ATPsyn β was possible located on the surface of some of these circulating cells in shrimp. This result was strengthened by the localization of ATPsyn β on epithelial cells of shrimp gill in our study by immunofluorescence assay and immunogold labeling technique. Furthermore, from the viewpoint of astakine receptor, ATP synthase enzyme complex was likewise detected only on the surface of HPT cells and not on any hemocytes in crayfish, because the expression of crayfish astakine was found to be restricted to the blood cells lineage [10]. While shrimp astakine mRNA is expressed in multi-tissues and organs, such as eyestalk, subcuticular epithelium, gills, heart, hepatopancreas, lymphoid organ, intestine, muscle, nerve and hematocytes [18,19]. Thus, there might have some circulating hemocytes with functional characteristics as hematopoietic stem cell in shrimp, which presented cell surface ATPsyn β. However, further investigation needed before any postulation on the difference of molecular mechanisms involved in the hematopoiesis between crayfish and shrimp.

It has been reported that F_1_F_O_ ATP synthase on endothelial cell surface is actively involving in ATP synthesis in human tumor, therefore, the generally accepted concept of ATP synthesis as a strict intracellular process now appears questionable [20]. However, it’s unknown for the presence of cell surface F_1_F_O_ ATP synthase and its roles in ATP synthesis in crustaceans. We speculate that the whole ATP synthase complex is present on the cell surface since its core component, the β subunit, has been confirmed to localize on cell surface. We quantified a detectable cell surface ATP production in shrimp hemolymph which indicated that cell surface ATP synthase is active in shrimp. Moreover, a significant suppressive effect on ATP synthesis activity on the cell surface has been demonstrated after WSSV infection. It’s well known that WSSV is an extremely virulent pathogen affecting various shrimps with a very rapid breakout [1]. Such rapid replication of WSSV in host cells will lead the quickly consuming of host ATPs and impact other energy-dependent biological function of the host cells, and eventually result in cell death [21].

On the other hand, the involvement of ATPsyn β, and its ligand astakine in hematopoiesis have been reported in crayfish, and in shrimp *Penaeus monodon* [22,23] and *L. vannamei* [19]. In our recently research, we have also identified astakine in *L. vannamei* (LvAST) and the envelope protein VP37 of WSSV competitively bound to BP53. LvAST and WSSV both likely use ATPsyn β on target cells as a receptor [24]. Given the role of ATPsyn β involves in hematopoiesis in crayfish [10], WSSV infection lead to host cell death possibly triggered by the competition between LvAST and the viral protein VP37 in binding to BP53. Further study is needed to clarify how the cellular process is carried through in host during WSSV infection.

In the time course analysis of *bp53* expression in responses to WSSV infection *bp53* expression was down-regulated in the early time post-injection in both WSSV challenged shrimp and the control shrimp. The penetration by the needle or virus stimulation appeared to induce some immune reaction, which in turn affected the normal physiological function of the shrimp, and the expression of *bp53* appeared down-regulation. At 8 h till 18 h post WSSV-challenge, the level of *bp53* expression in hemolymph was just slightly increased after dramatically decreased in first 4 h post injection. However, there was a significantly up-regulation of *bp53* gene expression from 12 h to 18 h post WSSV-challenge in shrimp gills, which might related to a different function of gill and hemocytes in systemic immune response to the WSSV. At 24 h post WSSV injection, the expression of *bp53* decreased both in hemolymph and gill, which might due to death of hemocytes and cells number decreased in WSSV-infected shrimp [25].

## Conclusions

Taken together, our results in the present study provide evidences in support of the hypothesis that ATPsyn β on host cell surface serves as a receptor for WSSV. Furthermore, the research revealed a possible molecular mechanism during WSSV infection, by which WSSV appears to disturb the host energy metabolism by interacting with host ATPsyn β. It also likely indicated the important role of cell surface ATP energy in WSSV’s binding and infection process.

## Materials and methods

### Shrimp

*L. vannamei* (Crustacea, Decapoda), approximately 8 g (fresh weight) and 6 to 8 cm long, were purchased from a local shrimp farm in Qingdao, Shandong Province, China. Shrimp were cultured in 80 L tanks (at 25°C) filled with air-pumped sea water. These shrimp were free of WSSV as tested by PCR.

Some naturally infected *L. vannamei* shrimp with WSSV were tested by PCR, and collected from a shrimp farm in Qingdao, Shandong Province, and were used in the study for cell surface ATP measurements.

### Virus source

A WSSV inoculum was prepared from WSSV-infected shrimp cephalothorax (2.16 × 10^3^ LD_50_/ml). Frozen infected tissue was homogenized in sterile HOPBS (288.8 mM NaCl, 2.7 mM KCl, 4.3 mM Na_2_HPO_4_, 1.4 mM KH_2_PO_4_) and centrifuged at 500 *g* for 10 min. The supernatant was filtered through a 0.45-μm Millipore filter and diluted 10^6^ times which was then used for inoculation to obtain WSSV-infected shrimp.

The intact WSSV viral particles from infected crayfish tissues were purified by differential centrifugation as described by Xie et al. [26]. The optical density of the purified sample was measured at 600 nm wavelength using a spectrophotometer. A formula C (virions/μl) = 3.34 × 10^8^ × OD600, which established by Zhou et al. [27] was used to convert the optical density of purified WSSV preparation into the virion concentration.

### BP53 polyclonal antibody preparation and specificity characterization

The purified recombinant BP53 (rBP53, GenBank accession number EU401720) was produced in *E.coli* expression system according to the method described previously [8], and was used as an antigen. A healthy New Zealand rabbit was subcutaneously injected with 200 μg purified rBP53 in 1 ml 0.9% NaCl as the primary immunization. Two additional booster injections were performed every 4 weeks. Ten days after the third injection, blood was collected from the immunized animal by exsanguination under general anesthesia and kept at room temperature for 3 h and then centrifuged for 10 min at 300 *g* to eliminate microclots and lipids. Serum was then collected and stored at-20°C. Pre-immune blood was collected from the ear artery of the same animal as control. The antiserum was incubated with lysates from *E. coli* Top10 cells with blank plasmid pBAD-gIIIB, which is produced by our lab, prior to applying for western blot and localization assays.

For the antibody specificity test, a western blot was carried out. Firstly, the lysates from *E. coli* Top10 cells with blank plasmid pBAD-gIIIB (as negative control), lysates of E. coli Top10 cells with recombinant plasmid pBAD-gIIIB-BP53 and gill membrane proteins extracted from WSSV-free *L. vannamei* were subjected to 12% SDS-PAGE, then transferred to PVDF membranes. The membrane was blocked with 10% BSA in PBS buffer containing 0.05% Tween 20 for 2 h at 37°C. Followed, 1:5000 rabbit anti-rBP53 antibody was added and incubated for 2 h at 37°C. After three washes with PBS contained 0.05% Tween 20, the membrane was incubated with 1:6000 horseradish peroxidase-conjugated goat anti-rabbit antibody (Invitrogen, USA) at 37°C for 1 hour. After wash, the signal was generated by BCIP/NBT substrate kit (Pierce, USA).

### Localization of BP53 in hemocytes by immunofluorescence assay

Hemolymph was collected from three WSSV-free *L. vannamei* shrimp using a 1-ml tuberculin syringe with a 26-gauge needle, 3.8% sodium citrate used as anticoagulant. Then, hemocytes were collected immediately by centrifugation at 600 *g* for 10 min, and suspended in 2 X L15 medium (GIBCO, China) with 20% (vol/vol) fetal calf serum (FCS) and 50 μg/ml antibiotics (10000U/ml Penicillin-10 mg/ml Streptomycin Solution). Then the hemocytes were incubated at 28°C for 1 h to allow cells to attach to the bottom of a petri dish. The hemocytes were then washed with PBS, divided into two groups and used for the following process. In control group, cells were permeabilized in absolute ethanol at RT for 3 min. All the cells were incubated with 10% bovine serum VI (Sigma) in PBS for 2 h and washed before incubation with rabbit anti-BP53 antibody (1:200) or pre-immune rabbit serum (1:200, negative control) at 28°C for 2 h. To test whether cells were permeabilized in each treatment, the cells were incubated with the antibody of cytoskeleton protein marker, actin, as the control. All cells were washed, and incubated with FITC-conjugated goat anti-rabbit IgG antibody (1:400, Invitrogen) and 0.01% (vol/vol) Evansblue for 1 h at 28°C. The nuclei were stained with DAPI. After final washes, cells were visualized under a confocal laser scanning microscopy (Nikon A1, Japan) at 425 nm and 488 nm wavelength correspondingly.

### Subcellular localization of BP53 in *L. vannamei* gills

#### Immunofluorescence assay

Gill secondary filaments tissues were removed from WSSV-free shrimp *L. vannamei* and immediately fixed in 4% formaldehyde fixation buffer for 12 h. The ultrathin paraffin sections were prepared and mounted on slides with coated poly-*L*-lysine. After de-waxing, sections were treated in 3% (vol/vol) methanol-H_2_O_2_ for 20 min, followed by high-pressure antigen retrieval in 0.5 M EDTA (pH 8.0) for 10 min. The indirect immunofluorescence assay was performed following the same procedure as described above.

#### Immunogold labeling technique

The gill filaments tissues were removed from WSSV-free *L. vannamei* and immediately fixed in glutaraldehyde-osmium acid buffer. The tissues were then embedded in Spurr. Sectuibs (50 nm thick) were collected on carbon-coated nickel grids. Sections were incubated with 1% H_2_O_2_ for 30 min followed by washing in distilled H_2_O and 0.05% TBS-Tween 20 three times (5 min per wash). Non-antigenic sites were blocked by incubating with 1% (wt/vol) TBS-BSA for 2 h at 37°C. The grids were then incubated with 200 μg/ml rabbit anti-rBP53 antibody in blocking buffer at 37°C for 1 h. After three washes, the sections were incubated with 10-nm colloidal gold-conjugated goat anti-rabbit IgG (Sigma, USA) in 1:200 dilution with blocking buffer for 1 h at 37°C. The sections were then washed three times with TBST buffer, followed three washes with H_2_O. Then the sections were post-stained with 2% (vol/vol) aqueous uranyl acetate for 10 min at room temperature, and incubated with Pb citrate for an additional 2 min. Parallel controls were performed with pre-immune serum. The sections were observed under an electron microscope (JEOL JEM-1200EX, US).

### Cell surface ATP assay

The cell surface ATP assay were performed as described with modifications [20]. Hemocytes were collected by centrifuging WSSV-free or WSSV naturally infected shrimp hemolymph, which was a mix of 3 individuals in each group, at 600 *g* for 10 min with 3.8% sodium citrate as anticoagulant. Hemocytes were seeded into 24-well plates at a density of 1000 cells per well and cultured in 2 X L15 medium with 20% FCS at 28°C for 3 h. Cells from WSSV-free shrimp were divided into two experimental groups. One group was incubated with purified WSSV at 4°C for 1 h, which allowed the virus just bound on the cells surface and not go inside the cells. One group was used for the following measurement without WSSV incubation. All the cells above were washed by Hepes buffer (10 mM Hepes, 150 mM NaCl, pH7.4), and incubated with 0.3 ml Hepes buffer containing 200 μM ADP, 20 mM potassium phosphate (Pi) and 2 mM MgCl_2_ at room temperature for 3 min. Supernatants were collected and centrifuged before assaying for ATP production by bioluminescent luciferase assay.

### Quantification of cell-surface ATP by bioluminescent luciferase assay

Aliquots of 100 μl supernatants from cell surface ATP assays as described above were analyzed using an ATP bioluminescence assay kit (Beyotime, China). In this study, only ATP is readily detected by the specific enzymatic reaction [20]. ATP generation was quantified by Varioskan flash multimode reader (Thermo scientific, Finland) and signals were recorded as RLU value. Data are expressed as picomoles of ATP produced per 10 cells relative to the standard determined under the same conditions with each experiment. All the experiments were carried out in triplicates, values reported are means of three replicates ± SD. One way ANOVA tests were used to test for statistically significant differences (p < 0.05).

### *bp53* expression upon WSSV infection by relative quantitative real-time PCR assay

*bp53* differential expression profiles in *L. vannamei* hemolymph and gill in response to WSSV infection was analyzed by relative quantitative real-time PCR. Shrimp was injected with 100 μL WSSV inoculum on the lateral side in the experimental group or with 100 μL sterile HOPBS in the control group with a 1-ml sterile syringe. Hemolymph and gill tissue from six shrimp per group per time point, were collected at 0 h, 2 h, 4 h, 8 h, 12 h, 18 h and 24 h post inoculation for RNA extraction.

Total RNA was extracted from the hemolymph using TRI Reagent (Invitrogen, USA) following the manufacturer’s instructions. 2 μg RNA was reversely transcribed with random 6 mers primer and the oligo (dT) primer using M-MLV reverse transcriptase (NEB, New Finland) to obtain first-strand cDNA. A 171-bp *bp53* gene fragment was amplified using primers 5’-TCT CTC TGA AGG ATG ATA C-3’ and 5’-GTG TGA AGC GGA AAA T-3’. Relative transcript quantities were calculated using the 2^−ΔΔCt^ method as described by Livak and Schmittgen [28] with β-actin as the reference gene amplified from the same samples. A 170-bp β-actin gene fragment was amplified using specific primers 5’ CGACCTCACAGACTACC 3’ and 5’ AGGACTTCTCCAGCG 3’. The PCR program was 95°C for 10 seconds followed by 40 cycles of 95°C for 6 s, 51°C for 30 s, and 72°C for 20 s. The real-time RT-PCR was performed in triplicate for the same sample in each experimental group. One way ANOVA tests were used to test for statistically significant differences (*p* < 0.05).

### RNAi-mediated silencing of the *bp53* gene

Double-stranded (ds) RNA corresponding to the *bp53* sequences was synthesized by *in vitro* transcription using the commercial RiboMAX™ T7 Express System kit (Promega, USA). Sense and antisense DNA templates for *in vitro* transcription were generated by PCR. The forward primers for both DNA templates were designed to contain T7 promoter sequence at the 5’ end (indicated by italics). The primers used for amplification of the sense DNA template were 5’ *GAT CCT AAT ACG ACT CAC TAT A*GG CCA TCT ATG TAC CTG CTG ATG ACT T 3’ and 5’ GCA GCC AAC TGT TCT GCC TTT TC 3’. The primers used for amplification of the antisense DNA template were 5’ GGC CAT CTA TGT ACC TGC TGA TGA CTT 3’ and 5’ *GGA TCC TAA TAC GAC TCA CTA TA*G CAG CCA ACT GTT CTG CCT TTT C 3’. Plasmid pBAD-gIIIA-BP53, which encodes the full-length cDNA of the *bp53* gene, was used as a template for PCR. An unrelated dsRNA corresponding to the green fluorescence protein (GFP) gene was prepared as a control as previously described [29].

Test shrimp (30 shrimp per group) were injected intramuscularly in the fourth or fifth abdominal segment with *bp53*-specific dsRNA (20 μg) or GFP-specific dsRNA (20 μg) dissolved in 100 μl of 150 mM NaCl solution using a 1-ml tuberculin syringe with a 26-gauge needle. Control shrimp were injected with 150 mM NaCl. Hemolymph (200 μl) was collected from three shrimp into 200 μl pre-cooled anti-coagulant solution (AC-l) (0.45 M NaCl, 0.1 M glucose, 30 mM sodium citrate, 26 mM citric acid, 10 mM EDTA, pH 4.6) [30] at 0 h, 10 h, 24 h, 33 h, and day 2 to day 5.

### RT-PCR analysis for *bp53* knock down

Total RNA was extracted from the hemolymph using TRI Reagent (Invitrogen, USA) following the manufacturer’s instructions. RNA was quantified by spectrophotometer, and 100 ng RNA of each sample was used for one-tube RT-PCR (Roche, USA). For *bp53* gene, the following primers were used: 5’ ATT TCT TTC CAG AGC CCT G 3’ and 5’ GGTATTGCCGAGTTGGGT 3’. Elongation factor (EF) was used as an internal control with the following primers (5’ GGT GCT GGA CAA GCT GAA GGC 3’ and 5’ CGT TCC GGT GAT CAT GTT CTT GAT G 3’). The PCR conditions for *bp53* and *ef* were as follows: 50°C for 30 min, 94°C for 2 min, followed by 28 cycles of 94°C for 10 s, 55°C for 30 s, and 68°C for 45 s, with a final extension at 68°C for 7 min. The PCR products of *bp53* (482 bp) and *ef* (187 bp) were analyzed by 1.2% agarose gel electrophoresis.

## Abbreviations

WSSV: White spot syndrome virus
VOPBA: Virus overlay proteins binding assay

## References

[1] Leu JH, Yang F, Zhang X, Xu X, Kou GH, Lo CF (2009). Whispovirus. Curr Top Microbiol Immunol.

[2] Smith AE, Helenius A (2004). How viruses enter animal cells. Science.

[3] Wang H-C, Wang H-C, Leu J-H, Kou G-H, Wang AHJ, Lo C-F (2007). Protein expression profiling of the shrimp cellular response to white spot syndrome virus infection. Dev Comp Immunol.

[4] Liang Y, Huang J, Song XL, Zhang PJ, Xu HS (2005). Four viral proteins of white spot syndrome virus (WSSV) that attach to shrimp cell membranes. Dis Aquat Organ.

[5] Chen LL, Lu LC, Wu WJ, Lo CF, Huang WP (2007). White spot syndrome virus envelope protein VP53A interacts with Penaeus monodon chitin-binding protein (PmCBP). Dis Aquat Organ.

[6] Li DF, Zhang MC, Yang HJ, Zhu YB, Xu X (2007). Beta-integrin mediates WSSV infection. Virology.

[7] Chen KY, Hsu TC, Huang PY, Kang ST, Lo CF, Huang WP (2009). Penaeus monodon chitin-binding protein (PmCBP) is involved in white spot syndrome virus (WSSV) infection. Fish Shellfish Immunol.

[8] Liang Y, Cheng JJ, Yang B, Huang J (2010). The role of F_1_ ATP synthase beta subunit in WSSV infection in the shrimp Litopenaeus vannamei. Virol J.

[9] Sritunyalucksana K, Wannapapho W, Lo CF, Flegel TW (2006). PmRab7 is a VP28-binding protein involved in white spot syndrome virus infection in shrimp. J Virol.

[10] Lin X, Kim YA, Lee BL, Soderhall K, Soderhall I (2009). Identification and properties of a receptor for the invertebrate cytokine astakine, involved in hematopoiesis. Exp Cell Res.

[11] Zhan W, Wang X, Chi Y, Tang X. The VP37-binding protein F1ATP synthase β subunit involved in WSSV infection in shrimp Litopenaeus vannamei. Fish Shellfish Immunol. 2013;34:228-35. 10.1016/j.fsi.2012.10.019 23108256

[12] Das B, Mondragon MO, Sadeghian M, Hatcher VB, Norin AJ (1994). A novel ligand in lymphocyte-mediated cytotoxicity: expression of the beta subunit of H+ transporting ATP synthase on the surface of tumor cell lines. J Exp Med.

[13] Fongsaran C, Jirakanwisal K, Kuadkitkan A, Wikan N, Wintachai P, Thepparit C (2014). Involvement of ATP synthase beta subunit in chikungunya virus entry into insect cells. Arch Virol.

[14] Paingankar MS, Gokhale MD, Deobagkar DN (2010). Dengue-2-virus-interacting polypeptides involved in mosquito cell infection. Arch Virol.

[15] Söderhall I, Bangyeekhun E, Mayo S, Söderhäll K (2003). Hemocyte production and maturation in an invertebrate animal; proliferation and gene expression in hematopoietic stem cells of Pacifastacus leniusculus. Dev Comp Immunol.

[16] Johansson Mats WKP, Sritunyalucksana K, Söderhäll K (2000). Crustacean haemocytes and haematopoiesis. Aquaculture.

[17] Giulianini PG, Bierti M, Lorenzon S, Battistella S, Ferrero EA (2007). Ultrastructural and functional characterization of circulating hemocytes from the freshwater crayfish Astacus leptodactylus: cell types and their role after in vivo artificial non-self challenge. Micron.

[18] van de Braak CB, Botterblom MH, Liu W, Taverne N, van der Knaap WP, Rombout JH (2002). The role of the haematopoietic tissue in haemocyte production and maturation in the black tiger shrimp (Penaeus monodon). Fish Shellfish Immunol.

[19] Liang GF, Liang Y, Cheng JJ, Zhang SC, Huang J (2012). Cloning, full-length sequence analysis and prokaryotic expression of astakine gene of Litopenaeus vannamei. Progress in Fishery Sciences.

[20] Moser TL, Kenan DJ, Ashley TA, Roy JA, Goodman MD, Misra UK (2001). Endothelial cell surface F1-F0 ATP synthase is active in ATP synthesis and is inhibited by angiostatin. Proc Natl Acad Sci U S A.

[21] Mowery YM, Pizzo SV (2009). The antitumorigenic trifecta. Blood.

[22] Söderhall I, Kim Y-A, Jiravanichpaisal P, Lee S-Y, Söderhall K (2005). An ancient role for a prokineticin domain in invertebrate hematopoiesis. J Immunol.

[23] Hsiao CY, Song YL (2010). A long form of shrimp astakine transcript: molecular cloning, characterization and functional elucidation in promoting hematopoiesis. Fish Shellfish Immunol.

[24] Liang G, Liang Y, Xue Q, Lu J, Cheng J, Huang J (2015). Astakine LvAST binds to the beta subunit of F-ATP synthase and likely plays a role in white shrimp Litopeneaus vannamei defense against white spot syndrome virus. Fish Shellfish Immunol.

[25] Chen IT, Aoki T, Huang YT, Hirono I, Chen TC, Huang JY (2011). White spot syndrome virus induces metabolic changes resembling the warburg effect in shrimp hemocytes in the early stage of infection. J Virol.

[26] Xie X, Li H, Xu L, Yang F (2005). A simple and efficient method for purification of intact white spot syndrome virus (WSSV) viral particles. Virus Res.

[27] Zhou Q, Qi Y-P, Yang F (2007). Application of spectrophotometry to evaluate the concentration of purified white spot syndrome virus. J Virol Methods.

[28] Livak KJ, Schmittgen TD (2001). Analysis of relative gene expression data using real-time quantitative PCR and the 2 (−Delta Delta C (T)) method. Methods.

[29] Saksmerprome V, Charoonnart P, Gangnonngiw W, Withyachumnarnkul B (2009). A novel and inexpensive application of RNAi technology to protect shrimp from viral disease. J Virol Methods.

[30] Söderhall K, Smith VJ (1983). Separation of the haemocyte populations of Carcinus maenas and other marine decapods, and prophenoloxidase distribution. Dev Comp Immunol.

